# Suicide with psychiatric diagnosis and without utilization of psychiatric service

**DOI:** 10.1186/1471-2458-10-431

**Published:** 2010-07-22

**Authors:** Yik-wa Law, Paul WC Wong, Paul SF Yip

**Affiliations:** 1 Department of Social Work and Social Administration, The University of Hong Kong, Pokfulam, Hong Kong SAR, China; 2 HKJC Centre for Suicide Research & Prevention, The University of Hong Kong, 3B, No. 2 University Drive, Pokfulam, Hong Kong SAR, China

## Abstract

**Background:**

Considerable attention has been focused on the study of suicides among those who have received help from healthcare providers. However, little is known about the profiles of suicide deceased who had psychiatric illnesses but made no contact with psychiatric services prior to their death. Behavioural model of health service use is applied to identify factors associated with the utilization of psychiatric service among the suicide deceased.

**Methods:**

With respect to completed suicide cases, who were diagnosed with a mental disorder, a comparison study was made between those who had (contact group; n = 52; 43.7%) and those who had not made any contact (non-contact group; n = 67; 56.3%) with a psychiatrist during the final six months prior to death. A *sample *of 119 deceased cases aged between 15 and 59 with at least one psychiatric diagnosis assessed by the Structured Clinical Interview for DSM-IV-TR (SCID I) were selected from a psychological autopsy study in Hong Kong.

**Results:**

The contact and non-contact group could be well distinguished from each other by "*predisposing*" variables: age group & gender, and most of the "*enabling"*, and "*need" *variables tested in this study. Multiple logistic regression analysis has found four factors are statistically significantly associated with non-contact suicide deceased: (i) having non-psychotic disorders (OR = 13.5, 95% CI:2.9-62.9), (ii) unmanageable debts (OR = 10.5, CI:2.4-45.3), (iii) being full/partially/self employed at the time of death (OR = 10.0, CI:1.6-64.1) and (iv) having higher levels of social problem-solving ability (SPSI) (OR = 2.0, CI:1.1-3.6).

**Conclusion:**

The non-contact group was clearly different from the contact group and actually comprised a larger proportion of the suicide population that they could hardly be reached by usual individual-based suicide prevention efforts. For this reason, both universal and strategic suicide prevention measures need to be developed specifically in non-medical settings to reach out to this non-contact group in order to achieve better suicide prevention results.

## Background

Retrospective psychological autopsy studies on completed suicides found that 60 to 90 percent of the suicide cases suffered from psychiatric illnesses prior to death [1-4]. However, a large proportion of suicides, ranging from 50 to 75 percent of suicides did not make contact with mental health services prior to their deaths [1,3-9]. This phenomenon has been suggested as one of the significant reasons of why suicide prevention efforts targeted on people with mental illnesses, i.e. medical treatment for people with depression, have been very limited in reducing suicide rates [10,11]. Therefore, in order to further reduce suicide rates, we must consider offering effective help not only to those currently being treated by our healthcare system, but also those who are currently not receiving care (namely the non-contact group). Unfortunately, many record-based studies on completed suicides can only study the psychiatric and psycho-social profiles of suicide cases that are known to the healthcare system [5,12-14]. Little was known about the profiles of the non-contact group as well as the factors surrounding their deaths [15]. It is imperative to investigate the characteristics and profiles of the non-contact group using a psychological autopsy methodology.

Among the paucity of studies that have investigated the non-contact group, some findings are noteworthy. Booth and Owens' review paper on five record-based studies that investigated the non-contact group comprised between 65 to 86 percent of all suicides and that males were significantly less likely than females to seek help [16]. Another study using linked population-based data in Taiwan showed that a much lower proportion of those committing suicide had received mental healthcare services within one year prior to their death (22.2% of 19 426 suicides). Those who had made no contact with a mental service professional tended to be male and aged over 55 [17]. Specifically, those who committed suicide by poisoning from charcoal burning in Taiwan had significantly fewer contacts with psychiatrists (18%) than those suicides by hanging (25%) and poisoning (23%) [18].

Owens and colleagues investigated the psychiatric and psycho-social aspects of the non-contact suicide group by comparing them with live controls [19]. They found the factors which predicted non-contact suicides were remarkably consistent with what was already known about suicides in general such as a previous attempted suicide, current or past mental illnesses, social and interpersonal problems. They also found that there was a markedly lower rate of mental illness (68%) among the non-contact suicide group than that of other psychological autopsy studies (90%). The authors, however, failed to consider mental illness as a major necessity in seeking care. Without considering mental illness as a *need *as well as a primary reason to seek mental health services and controlling this as the major variable in both suicide and live control groups, they were actually non-compatible in terms of the nature of the "*need*" of seeking help for medical treatment. In accordance with the Behaviour Model of access to medical care by Andersen [20], the "perceived" or "evaluated" need of a person should be considered as a precedent factor of whether or not it is important and a strong enough motive to seek professional help [20]. Therefore, it is crucial to gain a better understanding about psychiatric service-use among suicides with mental illness(es), before we suggest whether or not suicide rates can be reduced by improved psychiatric care [21,22].

The basic assumption of the behavioural model is that access to health care is a rational decision-making process during which "People's seeking healthcare services is a function of their predisposition to use those service factors, which enable or impede that use and the need for care" (pg. 1) [20]. 'Predisposing characteristics', for instance, include age, gender, genetic and psychological characteristics, social structure (i.e. education, ethnicity, social networks, interactions and culture) as well as health beliefs, values and general knowledge about health care and services. These are all important determinants that can directly affect one's perception of need for and the actual use of health services. "Enabling resources" are generally referring to the income and wealth available to individuals to pay for services, or if they have health insurance coverage, regular source of care, and travel time to and waiting time for care [23]. It is known that unmet need among people with mental illness for mental health service is likely associated with disadvantaged groups, such as those with low incomes, and those without insurance [24]. Therefore, financial resources or means of getting financial support are the basic components that can facilitate one's access to healthcare services. The third important component is 'need' which itself has two dimensions: perceived need and evaluated need. The former is the perception of how people view and experience their own general health and functional state and whether or not they find their own problems severe enough to seek professional help (help-seeking) and be compliant to treatment. The later is assessed by professionals and may not agree with a person's perceived need. It is closely related to the type and amount of treatment provided to a person after they seek help from a medical provider [20,25,26]. Besides, types of psychiatric diagnoses may also explain different level of health service use. Research showed that people who suffered from psychotic disorders showed high levels of mental health service use as well as community-based health service use in comparing to those with non-psychotic disorders [27,28].

Based on the behavioural model, this study will investigate the psychiatric and psycho-social characteristics of suicide deceased who were diagnosed with mental illnesses but did not make contact with any psychiatrist. It is hypothesized that the contact & non-contact group of suicide can be distinguished by "*predisposing*", "*enabling*" & "*need*" variables as suggested by the behavioural model. We utilised the data collected from a psychological autopsy study of suicides in Hong Kong between 2003 and 2005 to test our hypothesis. Comparisons will be made between those who had (contact group) and those who had not (non-contact group) visited any psychiatrist during the final six months prior to death.

## Methods

All suicide cases from the psychological autopsy study were identified from deaths registered as "suicide" by the Coroner in Hong Kong and coded from X60 to X84 under the Tenth International Classification of Disease (ICD-10) of the World Health Organization [29]. The study investigated 150 Chinese suicide cases aged 15 to 59 against a comparison of 150 live controls between September 2003 and December 2005. The study was approved by the Institutional Review Board of the University of Hong Kong/Hospital Authority Hong Kong West Cluster (HKU/HA HKW IRB) and the Ethics Committee of the Department of Health, Hong Kong SAR. Details of the inclusion of subjects, procedures of data collection, and measures have been described in previous reports [2,30]. A short summary of the methodology is given below.

We identified informants of suicide cases via the Forensic Pathology Service, which manages the public mortuaries in Hong Kong, and the Coroner's Court. For the informants recruited through the Forensic Pathology Service, the psychological autopsy study was first introduced by forensic pathologists at the public mortuaries and then via telephone by the research team. For the informants recruited from the Coroner's Court, we approached them first by an invitation letter (sent by the Coroner's Court) and followed by a telephone call by the research team.

The "evaluated need" for psychiatric service was defined as having at least one psychiatric diagnosis. Retrospective psychiatric diagnosis was assessed using the SCID-DSM-IV-TR (Structured Clinical Interview for Diagnostic and Statistical Manual of Mental Disorders - Forth Edition - Test Revision - Axis I disorders, American Psychiatric Association, 2000). The SCID-I-DSM-TR is the standard measurement of psychiatric disorders in psychological autopsy study [31].

### Sample

A total of 121 (80.7%) suicide deceased cases with at least one psychiatric diagnosis assessed by the SCID-I were then drawn from 150 of the suicide deceased group. Among the 121 cases, 52 (43.7%) had visited psychiatrists within the last six months prior to their death (contact group) whereas the other 67 cases (56.3%) did not do so (non-contact group). The next-of-kin of 2 cases did not have this information, therefore, a total of 119 valid cases were used for data analysis.

### Measures

#### Dependent Variables: Contact and Non-contact with psychiatric service

In the semi-structured interview, the next-of-kin of the suicide deceased were asked to retrospectively report the medical treatment history of the deceased. Those who had made contact with a psychiatrist within six months prior to their suicide were grouped under the "contact group" and those who hadn't made any contact were grouped under the "non-contact group".

#### Independent variables

The following three groups of independent variables were used to differentiate the contact from the non-contact group:

*Predisposing *- includes demographics and socio-economic characteristics such as age group, gender, marital status, education level, and years of living in Hong Kong, living arrangement and psychological characteristics. Impulsive-state behaviour was measured by the Impulsivity Rating Scale (IRS [32]).

*Enabling *- includes all indicators to show one's level of financial resource and/or potential resources from their networks which could either facilitate or impede his/her access to healthcare services. These variables are employment status, income level, unmanageable financial debts and bankruptcy, and living arrangement (i.e. whether the deceased lived with others or lived alone), social support and social problem solving skills as the indicators of potential resources available from their support networks. Social support was evaluated in terms of three aspects - the size of the social support network based on the number of close family members, extensive relatives and friends whom the subjects were able to rely on when dealing with life problems, frequency of social activities within the final month before the suicide, and social support content in terms of emotional, instrumental, informational and appraisal support. Informants of the deceased were asked to rate these four areas from scenario-based questions which determined the accessibility of support within the deceased's social network. Social problem-solving ability was measured by a shortened 8-item Social Problem-Solving Inventory (SPSI [33]) which was divided into four constructs: problem orientation, generation of alternative solutions, decision-making and solution implementation & verification.

*Need *- looks at the types of psychiatric diagnosis of SCID-I along with two additional Axis-I disorders - pathological gambling and intermittent explosive disorder. In this study we only identified on the 'evaluated need' since we could not measure the subjective 'perceived need' of the suicide deceased retrospectively. The evaluated need is divided into psychotic and non-psychotic disorders in according to the classifications of DSM-IV-TR (SCID I).

*Circumstances of suicide *- these are indicated by suicide intent (Beck Suicide Intent Scale-SIS [34]), suicide method and previous suicide attempt. The samples were divided into groups with low intent and high intent using a cut-off value (SIS = 5.5 for 7 items) [35].

#### Statistical Analysis

The SPSS-PC software version 16.0 statistical package was used to perform the statistical analysis. The age distribution and gender ratio of the total number of suicides aged 15-59 (n = 879) occurred in 2003 were used to compare with that of the 150 suicide cases and no significant difference was detected between the two samples [2]. A comparison was then conducted between the contact group and non-contact group with respect to the above mentioned independent variables through descriptive statistical analyses. A chi-square test and *t *test were used to examine the level of significance of the difference (p < .05) between these two groups. Those variables found to be significant were subsequently tested by a multi-variate logistic regression model in which a step-wise elimination method was used to create a stable model.

## Results

The mean age of the 119 suicide cases with at least one SCID diagnosis was 39.8 (S.D. = 11) years and the sample gender ratio between males and females was 1.5:1.

### Use of Psychiatric, Physical and Other Services for Emotional Problems

Among the 119 cases with at least one SCID diagnosis, 21 (17.4%) had made use of both psychiatric services and consulted a doctor for physical problems within six months prior to death, as well as being treated for mental health problems by other professions, i.e. clinical psychologist/social workers. But 14 (11.6%) cases had received no help at all. Compared with the contact group, more cases of the non-contact group had made contact with physicians (n = 44; 68.8%; X^2 ^= 3.00; p = .083) but the result was not significant. Moreover, only 27 cases (40.1%) in the non-contact group had sought treatment for their mental health problems. The percentage of those who had ever received treatment for mental health problems was significantly lower than that of the contact group (n = 39; 75%; X^2 ^= 13.71; p < = .000) (Table 1).

**Table 1:** Use of psychiatric, physical health and other services for mental health problems among the 121 cases with at least one SCID diagnosis

Visited a psychiatrist within the last 6 months		Contact (n = 52)	Non-contact (n = 67)	Missing (n = 2)	Total (N = 121)
Was the deceased under the care of a doctor in the last 6 months (X^2 ^= 3.00; df = 1; p = .083)	Y	27 (38.0%)	44 (62.0%)	6 (4.9%)	71 (61.7%)
	
	N	24 (54.5%)	20 (45.5%)		44 (38.3%)
					
					*Sub-total*: 115 (100%)

**Sub-total**		**51 (42.2%)**	**64 (52.9%)**		**121 (100%)**

Had the deceased been treated for mental health problems in any setting, i.e. clinical psychology or social services (X^2 ^15.4; df = 1; p > .000)	Y	40 (59.7%)	27 (40.3%)	3 (2.5%)	67 (56.8%)
	
	N	12 (23.5%)	39 (76.5%)		51 (43.2%)
					
					*Sub-total*: 118 (100%)

**Sub-total**		**52 (43.0%)**	**66 (54.5%)**		**121 (100%)**

Note: Among the 121 suicide cases, 21 (17.4%) had made use of all three types of services: psychiatric, physical and other psychological/social services while another 14 (11.6%) had received no help at all.

### "Predisposing" variables

Table 2 shows the comparative results between the contact and non-contact groups on their predisposing variables. The mean age of the contact group was 39.1 (S.D. = 11.8) and the non-contact group was 40.4 (S.D. = 10.2). A significant difference in the age group 35 to 44 was found between the contact and non-contact group (15.4% vs 40.3%; X^2 ^= 9.68, p = .046). There were significantly more cases of males (n = 46; 68.7%) in the non-contact than in the contact group (n = 26; 50%). The male to female ratio in the contact group was 1:1, while in the non-contact group it was 2.2:1. The two groups did not differ in terms of their marital status; years of living in Hong Kong; whether or not they lived alone, with family or others; and education level. No difference was found in terms of impulsivity levels.

**Table 2:** A comparison between the contact and non-contact group by predisposing variables

Predisposing variables	Contact gp.	Non-contact gp.	df	X^2^	t	P-value
Number of cases	52 (43.7%)	67 (56.3%)				

Gender			1	4.26		.039*
Male	26 (50.0%)	46 (68.7%)				
Female	26 (50.0%)	21 (31.3%)				

Age group			4	9.68		.046*
15-24	7 (13.5%)	6 (9.0%)				
25-34	14 (26.9%)	10 (14.9%)				
35-44	8 (15.4%)	27 (40.3%)				
45-54	18 (34.6%)	17 (25.4%)				
55+	5 (9.6%)	7 (10.4%)				

Marital status			6	9.48		.148
Single (never married)	26 (50%)	22 (32.8%)				
Married (living with spouse)	19 (36.5%)	28 (41.8%)				
Married (not living with spouse)	4 (7.7%)	2 (3.0%)				
Separated	0 (0.0%)	1 (1.5%)				
Divorced	2 (3.8%)	6 (9.0%)				
Widowed	0 (0.0%)	1 (1.5%)				
Defacto	1 (1.9%)	7 (10.4%)				

Education level			4	3.34		.502
University/post-secondary	8 (15.4%)	7 (10.4%)				
Senior secondary	16 (30.8%)	25 (37.3%)				
Junior secondary	13 (25.0%)	23 (34.3%)				
Primary	14 (26.9%)	11 (16.4%)				
Never been educated	1 (1.9%)	1 (1.5%)				

Yrs. of living in Hong Kong			1	1.42		.234
Less than 7 yrs.	4 (7.8%)	2 (3.0%)				
More than 7 yrs.	47 (92.2%)	65 (97.0%)				

Living arrangement			3	1.75		.627
Lived alone	11 (21.2%)	12 (17.9%)				
With family members	40 (76.9%)	52 (77.6%)				
With friends	0 (0.0%)	2 (3.0%)				
With others	1 (1.9%)	1 (1.5%)				

Level of impulsivity					-.22 -.22	.826 .829
Mean	5.02 (S.D. = 4.91)	5.22 (S.D. = 4.35)				

* p-value < .05

Note: According to the HK government, for those live more than 7 yrs. in HK are considered as residents.

### "Enabling" variables

Table 3 shows that the two groups were distinctly different from each other based on income level, employment status and condition of financial debts. The non-contact group tended to be fully or partly employed (n = 36; 55.4%; X^2 ^= 8.22; p = .016), and they comprised a higher percentage in terms of the level of income (HK$7K or more) (n = 25; 40.3%) than the contact group (n = 7; 14.9%; X^2 ^= 8.343; p = .004). But, the non-contact group had greater problems relating to financial debts (n = 29; 43.9%; X^2 ^= 16.71; p < .000) than the contact group (n = 5; 9.6%). They did not differ in terms of the size, frequency and content of their social support network but rather on social problem-solving ability (SPRI). The non-contact group appeared to have a higher mean of SPRI (Mean = 21.94; S.D. = 8.11; *t *= -2.96; p = .004) compared to the contact group (Mean = 17.50; S.D. = 8.10; *t *= -2.95; p = .004).

**Table 3:** A comparison between the contact and non-contact group by enabling, evaluated need and circumstance of death variables

Enabling, evaluated need & circumstance of death variables	Contact gp.	Non-contact gp.	df	X^2^	T	P-value
Number of cases	52 (43.7%)	67 (56.3%)				

Income level			1	8.34		.004*
Less than HK$7K	40 (85.1%)	37 (59.7%)				
HK$7K or more	7 (14.9%)	25 (40.3%)				

Employment status			2	8.22		.016*
Full/part-time/self-employed	15 (31.2%)	36 (55.4%)				
Unemployed/quitted	24 (50.0%)	25 (38.5%)				
Economically inactive	9 (18.8%)	4 (6.2%)				

Financial debts/bankruptcy			1	16.71		.000*
Debts/broke	5 (9.6%)	29 (43.9%)				
No debts/not broke	47 (90.4%)	37 (56.1%)				

Social problem-solving ability						
Mean	17.30 (S.D. = 8.09)	21.94 (S.D. = 8.11)			-2.96 -2.96	.004* .004*

Social support						
Size of social support	3.43 (S.D. = 2.86)	3.69 (S.D. = 2.37)			-.49 -.48	.623 .630
Frequency of social activities	6.15 (S.D. = 4.88)	7.90 (S.D. = 5.38)			-1.60 -1.62	.114 .110
Social support content	2.99 (S.D. = 1.13)	3.22 (S.D. = .95)			-1.08 -1.07	.281 .288

SCID diagnosis			1	22.0		.000*
Psychotic disorder(s)	23 (44.2%)	5 (7.5%)				
Non-psychotic disorder(s)	29 (55.8%)	62 (92.5%)				

Suicide Intent Scale						
Mean	6.88 (S.D. = 3.22)	9.52 (S.D. = 4.02)			-3.85 -3.96	.000*

Level of suicide intent			1	9.85		.002*
Low (0-5.5)	21 (40.4%)	10 (14.9%)				
High (5.6-14)	31 (59.6%)	57 (85.1%)				

Previous suicide attempt(s)			1	5.01		.082
Yes	31 (59.6%)	28 (41.8%)				
No	19 (36.5%)	38 (56.7%)				

Suicide method			6	12.18		.058
Cutting/stabbing	2 (3.8%)	1 (1.5%)				
Drug overdose	3 (5.8%)	4 (6.0%)				
Jumping railway	0 (0.0%)	1 (1.5%)				
Jumping from a height	34 (65.4%)	25 (37.3%)				
Hanging	3 (5.8%)	7 (10.4%)				
Carbon monoxide poisoning by charcoal burning	8 (15.4%)	25 (37.3%)				
Others	2 (3.8%)	4 (6.0%)				

* p-value < .05

The term of "Economic inactive" refers to those people who are disabled, retired or home makers.

The term of "Financial debts" means unmanageable debts, the total amount of which is > (average monthly income - basic living expenses) × 48 months.

Psychotic disorders include Schizophrenia, Delusional disorder, Brief psychotic disorder & other psychotic disorders NOS; Non-psychotic disorders include Major Depression, Dysthymia, Bipolar, Alcohol dependence, Non-alcohol substance abuse, Panic disorder, Adjustment disorder, Anxiety disorder, Obsessive compulsive disorder, Pain disorder & Pathological gambling.

### "Need" variables

Compared with the contact group (n = 29; 55.8%), the non-contact group had a much higher percentage of non-psychotic disorders (n = 62; 92.5%; X^2 ^= 21.997; p > .000), which included diagnoses of: major depression, dysthymia, bipolar disturbances, alcohol dependency, non-alcohol substance abuse, panic disorder, adjustment disorder, anxiety disorder, obsessive compulsive behavior, pain disorder and pathological gambling.

### Variables surrounding the circumstances of suicide

The two groups showed a significant difference on the mean of the suicide intent scale (SIS) (contact:6.88; S.D. = 3.22; *t *= -3.85; p < .000; non-contact:9.52; S.D. = 4.02; *t *= -3.96; p < .000). The non-contact group had a significantly larger percentage of high level SIS(> = 5.5) (n = 57; 85.1%) than the contact group (n = 31; 59.6%; X^2 ^= 9.86; p = .002). In addition, the non-contact group had equal numbers of cases where death occurred from carbon monoxide poisoning by charcoal burning (n = 25; 37.3%) and jumping from a height (n = 25; 37.3%) while the contact group had more cases where death occurred by the latter method (n = 34; 65.4%). Moreover, the non-contact group tended to have no record of previous suicide attempt (n = 38; 56.7%; X^2 ^= 5.01; p = .082) prior to death but the difference between the two groups was not statistically significant.

### Logistic Regression Analysis

A multiple logistic regression analysis was carried out and, four factors that were statistically significantly associated with non-contact suicide deceased cases: having non-psychotic disorders ("*evaluated need*") (OR = 13.5, 95% CI:2.9-62.9, p < .001), unmanageable debts ("*enabling*") (OR = 10.5, CI:2.4-45.3, p < .002), being full/partially/self employed at the time of death ("*enabling*") (OR = 10.0, CI:1.6-64.1, p < .015), and having a higher level of social problem-solving ability (SPSI) ("*enabling*") (OR = 2.0, CI:1.1-3.6, p < .018). They significantly predicted the likelihood of being the non-contact group of suicide cases. The model explains 54.2% of the variance between the contact and the non-contact group (Nagelkerke *R*^2 ^= 0.542). The results are reported in Table 4.

**Table 4:** Multivariate model ^a ^of associated factors comparing the contact group and non-contact group among deceased suicide cases

	O.R.	p <	95% CI	
			
			Lower	Upper
Financial debts/bankruptcy^1^ Debts/broke	10.5	.002*	2.42	45.29

SCID diagnosis^2^ Non-psychotic disorders	13.5	.001*	2.92	62.88

Social problem-solving ability	2.0	.018*	1.13	3.59

Employment Status^3^ Full/part-time/self-employed	10.0	.015*	1.56	64.15

* p-value < .05

a Reference categories: ^1^ No unmanageable debts/not broke, ^2^ psychotic disorders, ^3^ Unemployed or economic inactive

## Discussion

This study is the first of its kind to reveal the psychiatric and psycho-social characteristics of suicides with psychiatric illnesses but made no contact with psychiatric service prior to death (non-contact group). A larger proportion (56.4%) of suicide deceased investigated in this study was found in the non-contact group. Other than a few "*predisposing variables*", such as marital status, education level, years of living in Hong Kong, etc, the non-contact group was different from the contact group by variables of gender, and age group, and most of the "*enabling"*, "*need" *and circumstance of death variables tested in this study. Four major factors were found to statistically significantly associate with the non-contact group which were having non-psychotic disorders, unmanageable debts, being full/partially/self employed and having higher levels of social problem-solving ability.

### The proportion of the non-contact group

In societies where resources are limited, it is understandable that suicide deceased would have had considerable barriers of accessing to mental health services prior to death. A large scale of psychological autopsy study on suicides in China showed that among the 563 suicide cases with mental illness, only 13% (76/563) received psychiatric treatment [36]. While in Pakistan, a low to middle-income country, the use of psychiatric service was even much lower that only nine out of 100 suicide cases had ever received treatment for psychiatric disorders in the past [37]. In Hong Kong as one of the fast-growing economic cities in Asia, all residents are entitled to enjoy highly subsidized health care provided by public hospitals and health clinics which are basically tax-funded like many other ex-colonies in the British Commonwealth [38]. The charge for general outpatient consultation per visit is US$5.7- (HK$45) while specialist consultation is US$7.7 - 12.8 (HK$60-100). Comparatively, suicide cases in Hong Kong in terms of affordability would have had better chances of accessing to specialized healthcare services than that of those cases in developing and under-developed countries. Given such low-cost healthcare provision, the use of psychiatric service among suicides in Hong Kong was far from satisfactory since over half of the suicide deceased with psychiatric illnesses had not received any psychiatric care prior to their deaths.

### Variables of behavioural model associated with psychiatric service use

The non-contact group of the suicide deceased in this study was not found to be as similar in terms of their characteristics as suggested by other studies on people with mental illnesses. For example, in Canada, among those who had a DSM-III- diagnosed mental disorder, over 90% did not consult a psychiatrist during the previous year. The major determinants of seeking healthcare services were sex (female), age (under 45), severity of the illness and co-morbidity[39]. It was also found that unmet need for treatment was found to be greatest in traditionally underserved groups such as the elderly, racial-ethnic minorities, low-income people, those without insurance and residents of rural areas [24]. We will discuss each of the differences between the contact and non-contact group with suggested strategies for suicide prevention.

#### Pre-disposing variables

In other studies males in the youngest and oldest age groups were found to be least likely to have used mental health services prior to their suicide [16]. The non-contact group in this study was comprised predominantly of males (M:F 2.2:1), but tended to be more prevalent among adults aged between 25 and 44, which is the most productive period in one's life. The gender difference with respect to making use of health services is also commonly found in populations that suffer from mental illness [24,28]. This could be one of the most important factors contributing to the gender difference among suicides between males and females because the latter often are pro-active in their help-seeking behaviour when problems arise [40]. The similarities of the two groups in terms of their marital status, years of living in Hong Kong, living arrangements, education level and level of impulsivity suggested that the non-contact group was not particularly more susceptible than the contact group to any of these commonly known vulnerabilities which might hinder their access to the health service system.

#### Enabling variables

The results of this study further reveal that the non-contact group saw a significantly higher percentage of employed suicide victims (55.4%) who also had relatively higher levels of income compared with the contact group. Multiple logistic regression analysis showed that unmanageable debts (10.5) and being full/partially/self employed at the time of death were two important factors to associate with those in the non-contact group. Also, the non-contact group tended to have a higher level of social problem-solving ability than that of the contact group, and their social support in terms of size, frequency and content made no different from the contact group. These data suggests that the non-contact group tended to be more competent in securing jobs and resolving problems than the contact group. In terms of enabling resources, apparently the non-contact group was no less deprived than the contact group to have access for medical treatment. Unlike what Andersen's behavioural model suggests, individual enabling characteristics are not the crucial determinants to predict the use of psychiatric service among the suicide cases.

Suicides in Hong Kong and in other developed countries in Asia have been found to be strongly associated with socio-economic factors, such as individual financial debt problems [2,30,41], unemployment [42], and economic crises [43,44]. It is interesting to note that unmanageable debts not only associated with suicide deceased but also could predict their health service use pattern. Those with problems of unmanageable debts and other financial difficulties might not find psychiatric treatment useful even though at the same time they were having symptoms of mental illnesses. It could be that the beliefs and attitudes of the non-contact group on how they perceive their problem and its relevance of consulting a psychiatrist are crucial components of their actual service use. Despite some methodological limitations, it will be of an interest to examine whether or not psychiatric treatments can be seen as useful for the non-contact group of suicide.

#### Need variables

Having non-psychotic disorders (O.R. 23.71) rated by the SCID was the strongest predictor of making no contact with a psychiatrist. Those who had non-psychotic disorders were found to have made less use of psychiatric services than those who had psychotic disorders. A large scale 4-year study on mental health use among a population of 1.1 million people in Canada showed that intensive users of mental health services comprised 27.4% of patients in treatment for psychotic disorders but only 4.4% of those receiving treatment for non-psychotic disorders [45]. Moreover, the study also determined that people with psychotic disorders were also the ones with very high rates of mental healthcare use, including outpatient and community-based healthcare services. In fact, they were two to four times more likely than people with non-psychotic disorders to have obtained outpatient mental health services and had more than twice the number of contacts with these services [27]. Phillips et al found in their epidemiological survey in China that more than 70% of patients with psychotic disorders had received professional help while more than 88% of those suffered from non-psychotic mental disorders had never received any type of health services. The traditional mental healthcare system which mainly occupied by patients with psychotic disorders, was considered inadequately equipped to provide counselling services for those who suffered from depression and other less severe psychological problems [28]. On the other hand, psychiatric services may not be seen as helpful in resolving those problems faced by those who suffer from non-psychotic disorders, particularly among individuals with depression. As suggested by Takahashi, about 90% of suicide cases were diagnosed as depression-related in Japan and many were males who tended not to consult others when they were facing problems. They did so because they were afraid that it would become a negative factor in their job personnel evaluations if they admitted to having mental problems in the midst of a recession [44].

Apart from the evaluated need, the perceived need of people with suicidal behaviours was found to play an important role of determining the level of actual service use. The study of Pagura et al [46] showed that although live individuals who had suicide ideations and attempts tended to have higher proportions of help seeking and perceived need for health service use in comparing with those with mental disorders, large proportions of them (suicide attempters: 59%; individuals with suicide ideation: 76%) did not perceive any need for treatment. The authors explained that without perceived need of health services, the severity of evaluated need by itself might not promote help seeking. As to the suicide deceased group, perhaps the perceived need of psychiatric service is the missing link to explain on why a large proportion of suicide deceased group did not make use of the services. In view of the fact that data related to self-perception could hardly be collected from deceased samples, future studies on the perceived need can be considered to capture from individuals with nearly lethal suicide attempts [47].

### Circumstances of death

The non-contact group was found to have a significantly higher level of suicide intent and tended to choose carbon monoxide poisoning by charcoal burning which has a high lethality. This agrees with findings from a Taiwanese study in which suicide victims who died by charcoal burning were also found to have fewer healthcare contacts prior to their suicide [18]. It appeared that the non-contact group was showing obvious signs of mental illness and a high degree of suicidal intent while not seeking psychiatric services for their problems. What is worse is that because they had fewer suicide attempts, it provided no sign of risk to the people around them. These were indeed people in the high-risk group who allowed limited opportunities for clinical intervention.

## Limitations

This study has several limitations. The methodology of using data provided by informants of suicide deceased has been criticised as having problems of recall bias [48]. Even when the informants could recall accurate details of the deceased's life events, the data could still be unreliable due to factors including deliberately withholding information or distortions due to the informants' emotional state [48]. Besides, evidence suggested that all suicide cases were probably associated with abnormalities of psychiatric disorders which could hardly be detected through proxy information provided by informants [49]. The psychiatric diagnosis assessed retrospectively in psychological autopsy studies could have been underestimated. Given the limitation that we could not generate any findings about suicide cases' access to care though direct interviews with them, the psychological autopsy study design allows us to investigate the suicide deceased's use of health services retrospectively through the use of interviews with informants closest to them.

## Conclusions

This is the first study to delineate the psychiatric and psycho-social profile of suicide deceased cases with psychiatric illnesses beyond the reach of the mental healthcare system. Results show that the non-contact group was different from the contact group and actually represents a larger proportion of the suicide population. Their tendency to not contact with a psychiatrist prior to their suicide can be predicted by several factors such as suffering from non-psychotic disorders, having unmanageable debts, having a relatively stable employment, and a higher level of social problem-solving ability. Interestingly, most of the enabling factors associated with the non-contact group of suicide deceased are unexpectedly not consistent with those suggested by Andersen's behavioural model. It may be explained by the strong resistance or a lack of readiness of the suicide population in seeking help from psychiatric services for their mental health problems. They are the most difficult group to be reached by healthcare professionals and that makes individual-based suicide prevention efforts hardly effective.

To develop more effective suicide prevention measures in order to reduce suicide rates significantly, we must consider better ways to offer effective help not only to those who are known to the healthcare system, but also to those who are beyond the reach of existing services, especially among those who may currently be in the work force. It is still an open question as to whether or not advancements in psychiatric service can be correlated with a reduction in the suicide rate [22,50-52]. Apparently with a large proportion of suicide deceased people who had no contact with mental health services, this could hardly be the answer [52,53]. Moreover, this is particularly true in areas with a large population and limited resources, such as Asia, where nearly 600,000 of suicide deaths happen every year[54].

The restriction of access to suicide means should be widely adopted as a universal safety measure for suicide prevention. This includes limiting access to charcoal packs to prevent suicide by charcoal burning [55] as well as the installation of platform screen doors in railway stations [56]. These universal measures have been effective in reducing suicide deaths. In addition, innovative approaches for engaging people who are potentially at risk for suicide in various types of non-medical settings should be encouraged. For example, referrals to mental health services should be arranged for those who experience job-related stress or emotional problems at the work places. This is also true for people who encounter debt repayment difficulties during economic crises. Community-based suicide prevention programmes that aim to strengthen neighbourhood networks and encourage help-seeking behaviour should be promoted as well. Moreover, it is also worth investigating how to make use of innovative communication means, such as the internet or social media networks for delivering mental health literacy messages to high-risk groups. More research studies on the patterns and effectiveness of these innovative communication means for suicide prevention should also be emphasised. In conclusion, we must consider using multiple strategic approaches to reach those who are unlikely to seek traditional types of psychiatric services on their own and motivate them to get proper care for their mental disturbance. With additional research and understanding about this silent majority cohort, better strategic efforts to reach them can be developed for more promising suicide prevention results.

## Competing interests

The study was supported by the Hong Kong Jockey Club Charities Trust (which underwrote this research study via the Chief Executive's Community Project List 2002).

## Authors' contributions

YWL formulated the hypothesis, conducted the data analysis and wrote up initial drafts of this manuscript. PWCW, YWL and PSFY were involved in the design of the psychological autopsy study. PWCW and YWL were involved in the data collection. All authors edited and approved the final version.

## Pre-publication history

The pre-publication history for this paper can be accessed here:

http://www.biomedcentral.com/1471-2458/10/431/prepub
